# Prevalence and Ecological Role of *Streptococcus toyakuensis* in Saliva of Healthy Young Individuals

**DOI:** 10.1016/j.identj.2025.103987

**Published:** 2025-11-09

**Authors:** Nami Obayashi, Tomoaki Shintani, Nagisa Morihara, Miki Kawada-Matsuo, Toshinori Ando, Rie Miyata, Yuka Hayashi, Nanako Kataoka, Kotaro Tanimoto, Hiroyuki Kawaguchi, Hitoshi Komatsuzawa, Mikihito Kajiya

**Affiliations:** a Center of Oral Clinical Examination, Hiroshima University Hospital, Hiroshima, Japan; b Department of General Dentistry, Hiroshima University Hospital, Hiroshima, Japan; c Natural Science Center for Basic Research and Development, Hiroshima University, Hiroshima, Japan; d Department of Bacteriology, Graduate School of Biomedical and Health Sciences, Hiroshima University, Hiroshima, Japan; e Orthodontics and Craniofacial Developmental Biology, Graduate School of Biomedical and Health Sciences, Hiroshima University, Hiroshima, Japan

**Keywords:** *Streptococcus toyakuensis*, *Streptococcus mitis* subsep. m*itis*, Antimicrobial resistance, Oral microbiome, 16S rRNA gene next-generation sequencing

## Abstract

**Background and Objectives:**

Antimicrobial resistance (AMR) remains a critical global health concern. In 2022, *Streptococcus toyakuensis*, a newly identified species with potential multidrug resistance, was isolated from the blood of a patient with sepsis. This study aimed to explore the distribution of *S. toyakuensis* in the oral microbiome of healthy young individuals and to compare the bacterial community composition between the detection and non-detection groups.

**Methods:**

Sixty saliva samples were randomly collected from 356 healthy young individuals and analyzed using next-generation 16S rRNA sequencing for comprehensive microbial profiling. A taxonomic distribution analysis was performed to compare microbial diversity between *S. toyakuensis* detection and non-detection groups. Functional analysis identified differentially activated metabolic pathways.

**Results:**

*S. toyakuensis* was detected in 35 of 60 participants. Beta diversity analysis revealed a significant difference in microbial composition between the groups. Linear discriminant analysis effect size showed higher abundance of *Neisseria, Haemophilus, Campylobacter*, and *Capnocytophaga* in the detection group, while *Actinomyces* predominated in the non-detection group. Functional analysis identified 26 significantly different metabolic pathways, including the glyoxylate cycle and L-methionine biosynthesis superpathway.

**Conclusion:**

The high prevalence of *S. toyakuensis* in the oral microbiome of healthy young individuals highlights the need for further investigation into its role in AMR dissemination.

**Clinical Significance:**

The colonization of *S. toyakuensis* may influence the oral microbial ecosystem and metabolic activity, potentially facilitating the spread of AMR. Its detection in healthy individuals suggests a silent reservoir, underscoring the importance of oral microbiome surveillance in public health and infection control strategies.

## Introduction

Antimicrobial resistance (AMR) represents one of the most critical global health challenges, posing significant threats to public health and healthcare systems worldwide. In 2021, an estimated 4.71 million deaths were associated with AMR, of which 1.14 million were directly attributable to AMR.^1^ Furthermore, if left unaddressed, AMR is projected to cause up to 10 million deaths annually by 2050 and a cumulative economic loss of $100 trillion USD, as reported in *Tackling Drug-Resistant Infections Globally: Final Report and Recommendation*s.^2^ These alarming projections underscore the urgent need for in-depth research on the mechanisms of resistance and pathways through which resistant bacteria spread. The oral microbiome has been identified as a significant contributor to AMR and a potential reservoir for resistance genes. The oral microbiome harbors a higher density of resistance genes than the gut microbiome; however, its composition and role in AMR remain poorly understood.^3^ Oral bacteria have demonstrated the capability to spread beyond the oral cavity, colonizing distant sites such as the bloodstream, lungs, and gastrointestinal tract, and facilitating transmission to other individuals. Consequently, these bacteria serve as key mediators in the dissemination of resistance genes.^3^, ^4^, ^5^ Findings from long-term care facilities further underscore this concern, with methicillin-resistant *Staphylococcus aureus* detected in 7.9% of oral samples and non-antimicrobial-resistant bacteria identified in 17.8%.^6^

In 2022, a novel alpha-hemolytic *Streptococcus* strain resistant to β-lactams, macrolides, and quinolones was isolated from the blood of a 34-year-old patient with sepsis in Japan. This strain, designated as *Streptococcus toyakuensis sp. nov.* and belonging to the *Streptococcus mitis* group,^7^^,^^8^ has attracted attention due to its ability to acquire resistance genes through natural mutations and horizontal gene transfer. This bacterium exhibits resistance to β-lactams, macrolides, tetracyclines, and quinolones.^7^ The β-lactam resistance is believed to arise from intrinsic mutations in penicillin-binding proteins (PBPs)—particularly pbp1a, pbp1b, pbp2b, and pbp2x—that alter drug-binding affinity and reduce susceptibility. Similarly, quinolone resistance is associated with mutations in gyrA and parC, genes encoding the subunits of DNA gyrase and topoisomerase IV, respectively, which mediate quinolone activity. By contrast, macrolide and tetracycline resistance has been attributed to acquired resistance genes located on mobile genetic elements, such as transposons. Macrolide resistance is mediated by erm(B) and mef(A), which confer ribosomal target modification and efflux pump activity, respectively. Meanwhile, tetracycline resistance is associated with tet(M), which encodes a ribosomal protection protein. The presence of these resistance determinants suggests the potential for horizontal gene transfer among α-hemolytic streptococci, raising concerns regarding the dissemination of AMR within the oral microbiome. Although the clinical significance of this strain is evident, its distribution in the oral microbiome and its potential influence on microbial activity remain unknown.

To address this gap, we analyzed saliva samples from 60 young individuals using next-generation sequencing (NGS), discovering that 58.3% of the samples harbored *S. toyakuensis*. Functional pathway analysis revealed significant differences in microbial activity between groups with and without the organism. Although these findings do not directly establish its role in AMR mechanisms, they provide foundational insights into the prevalence and potential significance of *S. toyakuensis* within the oral ecosystem.

## Materials and methods

### Study population

This study builds on our previous study^9^ and adheres to the same inclusion and exclusion criteria. Data were collected from patients who underwent saliva testing at the Department of Orthodontics, Hiroshima University Hospital, between March 2022 and February 2024, with a specific focus on detecting *S. toyakuensis*. Verbal consent was obtained from all participants at the time of study enrollment. For participants aged <18 years, consent was obtained from a parent or legal guardian. Participants who had taken antibiotics or other medications within the 14 days prior to saliva testing were excluded. Individuals with congenital diseases, including oligodontia, were also excluded. Furthermore, those whose total volume of stimulated saliva was less than 2 mL were excluded due to insufficient sample volume for reliable bacterial quantification. At baseline, all participants underwent a clinical dental examination and provided stimulated saliva samples for testing.

This study adhered to the ethical guidelines of the Declaration of Helsinki and was approved by the Ethics Committee of Hiroshima University Hospital (approval number: epidemiology-2022-0205).

### Clinical oral examination

Participants' tooth count and oral hygiene status were assessed by a qualified dentist or dental hygienist. The Plaque Control Record was evaluated based on the O’Leary index.^10^ Plaque staining was applied to six surfaces of each tooth above the gingival margin (buccal, lingual, proximal, and distal surfaces) to visualize plaque deposits. The percentage of stained surfaces was calculated and recorded as the Plaque Control Record score. The total number of erupted teeth was defined as the total tooth count in the oral cavity.

### Saliva collection and analysis

Saliva samples were collected by instructing participants to chew tasteless paraffin gum, ensuring that at least one hour had elapsed since their last meal, beverage, or oral hygiene activity. The stimulated saliva flow rate (mL/min), pH, and buffer capacity were measured immediately after sample collection by trained personnel. The buffering capacity of the stimulated saliva was evaluated using the CAT21 Buf system (Morita Co., Osaka, Japan). For this analysis, 1 mL of saliva was mixed with dried lactic acid powder, and the final pH was measured after thorough mixing using a laboratory shaker. Saliva pH and buffering capacity were assessed using a compact pH meter (LAQUAtwin; HORIBA, Ltd., Kyoto, Japan).

### Bacterial count in saliva and detection of *Candida*

The bacterial count in saliva was measured using an Oral Bacterial Counter (Panasonic Healthcare Co. Ltd., Osaka, Japan). Fifty µL of saliva were placed in a disposable cup, and changes in impedance were measured and converted into bacterial concentration, expressed in colony-forming units per milliliter (CFU/mL), using the dielectrophoretic impedance measurement method.

*Candida species* were detected by spreading 20 µL of saliva onto CHROMagar *Candida* medium (CHROMagar, Paris, France), followed by incubation at 37°C for 48 hours under aerobic conditions. The results were considered positive if at least one colony was observed.

### Bacterial DNA extraction and 16S rRNA sequencing

Following saliva sample testing, bacterial genomic DNA was extracted from 60 saliva samples using the MagMax^TM^ Saliva gDNA Isolation Kit (Thermo Fisher Scientific, Waltham, MA, USA) and stored at −80°C until further analysis.

The extracted DNA was used as a template for polymerase chain reaction (PCR) amplification using the GoTaq Master Mix (Promega, Madison, WI, USA). Universal primers with Illumina adapter overhang sequences targeting the 16SrRNA region were used: V3-F (5′-TCGTCGGCAGCGTCAGATGTGTATAAGAGACAGCCTACGGGNGGCWGCAG-3′) and V4-R (5′-GTCTCGTGGGCTCGGAGATGTGTATAAGAGACAGGACTACHVGGGTATCTAATCC-3′). The PCR amplification protocol included an initial denaturation step at 94°C for 5 minutes, followed by 40 cycles of denaturation at 94°C for 30 seconds, annealing at 55°C for 30 seconds, and extension at 72°C for 30 seconds. The final extension was performed at 72°C for 5 minutes, and the samples were subsequently maintained at 4°C.

To confirm the presence of amplified bacterial DNA, PCR products were analyzed by 1.2% agarose gel electrophoresis. The gels were stained with ethidium bromide (0.5 µg/mL) and visualized under ultraviolet light illumination. Prior to amplification, the quality and purity of the extracted DNA were assessed using a NanoDrop spectrophotometer by evaluating A260/A280 and A260/A230 ratios. Only samples that met established thresholds for purity and concentration were used for PCR and subsequent analysis.

### Next-generation sequencing of salivary samples

Saliva samples confirmed to contain bacteria were subjected to comprehensive sequence analysis using NGS (Macrogen Japan Corp., Tokyo, Japan). Library preparation and sequencing were performed according to the company’s standard protocol, utilizing the Illumina MiSeq platform with 300 bp paired-end reads.

### Sequence data processing and diversity analysis

Sequence data were analyzed using Quantitative Insights into Microbial Ecology (QIIME2, version 2023.02). Low-quality reads and adapter sequences were removed, and paired-end reads from 60 samples were demultiplexed and imported into the QIIME2 platform. Sequence quality control and feature table construction were performed using the divisive Amplicon Denoising Algorithm 2 plugin.

Taxonomic distribution analysis was conducted using a pre-trained naïve Bayesian classifier trained on the SILVA database (version 138). Alpha diversity indices (Shannon, Faith’s phylogenetic diversity, observed features, and evenness) and beta diversity indices (Bray–Curtis and weighted UniFrac) were calculated using QIIME2.

### Statistical methods

Statistical analyses were performed using JMP Pro 18 (SAS Institute Inc., Cary, NA, USA) and QIIME2. Univariate analysis was performed to evaluate the differences between the *S. toyakuensis -*positive and *S. toyakuensis* -negative groups. For continuous variables, the Student’s t-test was used to analyze parametric data, whereas the Wilcoxon rank-sum test was used for assessing non-parametric data. Categorical variables were evaluated using Fisher’s exact test.

Beta diversity indices of the salivary microbiota were visualized using principal coordinate analysis (PCoA). Significant differences in alpha and beta diversity indices between the groups were evaluated using the Kruskal-Wallis test and pairwise permutational multivariate analysis of variance (PERMANOVA) within the QIIME2 framework. To control the false discovery rate and minimize the likelihood of type I errors in multiple comparisons, the Benjamin-Hochberg method was applied, and adjusted q-values were calculated.

Microbiome composition was visualized using QIIME2 (https://view.qiime2.org). Linear discriminant analysis effect size (LEfSe) was employed to identify taxa that characterized the differences between the groups. LEfSe utilized the Kruskal-Wallis rank-sum test (α=0.05) to detect significant features, followed by linear discriminant analysis (LDA) to estimate the effect sizes of these features.

Functional prediction of microbial communities was performed using Phylogenetic Investigation of Communities by Reconstruction of Unobserved States 2 (PICRUSt2). Predicted functions were statistically analyzed using Statistical Analysis of Metagenomic Profiles (STAMP), and significant differences between the groups were identified. Functions found to be significant were further investigated using the MetaCyc Metabolic Pathway Database (Metabolic Pathways From all Domains of Life) for detailed pathway analysis. A p-value of <0.05 was considered significant.

## Results

**Fig. 1:**
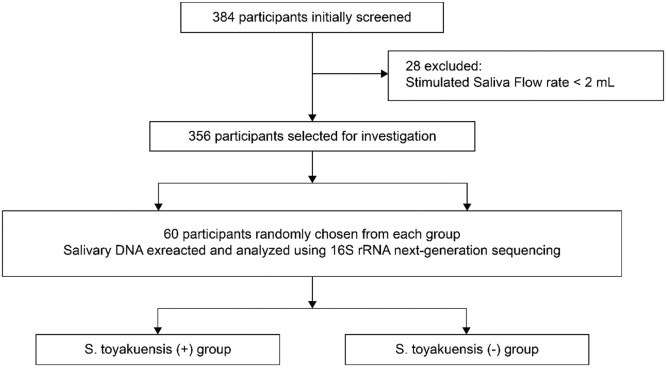
Patient selection and grouping flow chart.

A total of 384 participants were initially screened, of whom 356 met the inclusion criteria. Sixty individuals were randomly selected, and their saliva samples were analyzed using 16S rRNA-based NGS to identify all salivary bacterial species.^9^ Based on the sequencing results, 60 individuals were categorized into *S. toyakuensis* (+) and *S. toyakuensis* (−) groups (Figure 1).

The statistical characteristics of the two groups are presented in Table 1. *S. toyakuensis* was detected in 35 participants (58.3%). The relative abundance of *S. toyakuensis* was 0.0016% (interquartile range: 0.0007%-0.0051%). Univariate analysis revealed no significant differences between the groups in terms of age, sex, stimulated salivary flow rate (SSFR, mL/min), salivary pH, buffering capacity, tooth count, plaque control record, salivary bacterial count (CFU/mL), or positive rate of *Candida* species.

**Table 1:** Statistical characteristics (n = 60).

Characteristic		*S. toyakuensis* (+)	*S. toyakuensis* (−)	p-value
		(N = 35)	(N = 25)	
Age, year	Median [IQR]	11 [8-18]	12 [9.5-23]	0.3174*
Sex, no. (%)	Male	18 (69.2)	8 (30.8)	0.1878†
	Female	17 (50.0)	17 (50.0)	
SSFR, ml/min	Mean ±SD	1.203 ± 0.09	1.148 ± 0.41	0.6731‡
pH of SS	Median [IQR]	7.7 [7.5-7.8]	7.6 [7.4-7.8]	0.5893*
Buffer capacity of SS	Median [IQR]	6.6 [6.4-6.8]	6.7 [6.4-7.0]	0.4072*
No. of teeth	Median [IQR]	25.5 [24-28]	24 [23-28]	0.1223*
Plaque control record, %	Mean ±SD	55.44 ± 20.88	57.52 ± 16.33	0.6810‡
No. of oral bacteria, ×10^6^cfu/ml	Median [IQR]	88.9 [23.6-236.0]	103.0 [22.2-140.5]	0.7013*
*Candida albicans*, no. (%)	Positive	1 (2.9)	2 (8.0)	0.5653†

Statistical analyses were performed using the following methods:

⁎ Wilcoxon rank-sum test.

† Fisher’s exact test.

‡ Student’s t-test. Abbreviations: IQR, interquartile range; SD, standard deviation; SSFR, stimulated saliva flow rate; SS, stimulated saliva.

**Fig. 2:**
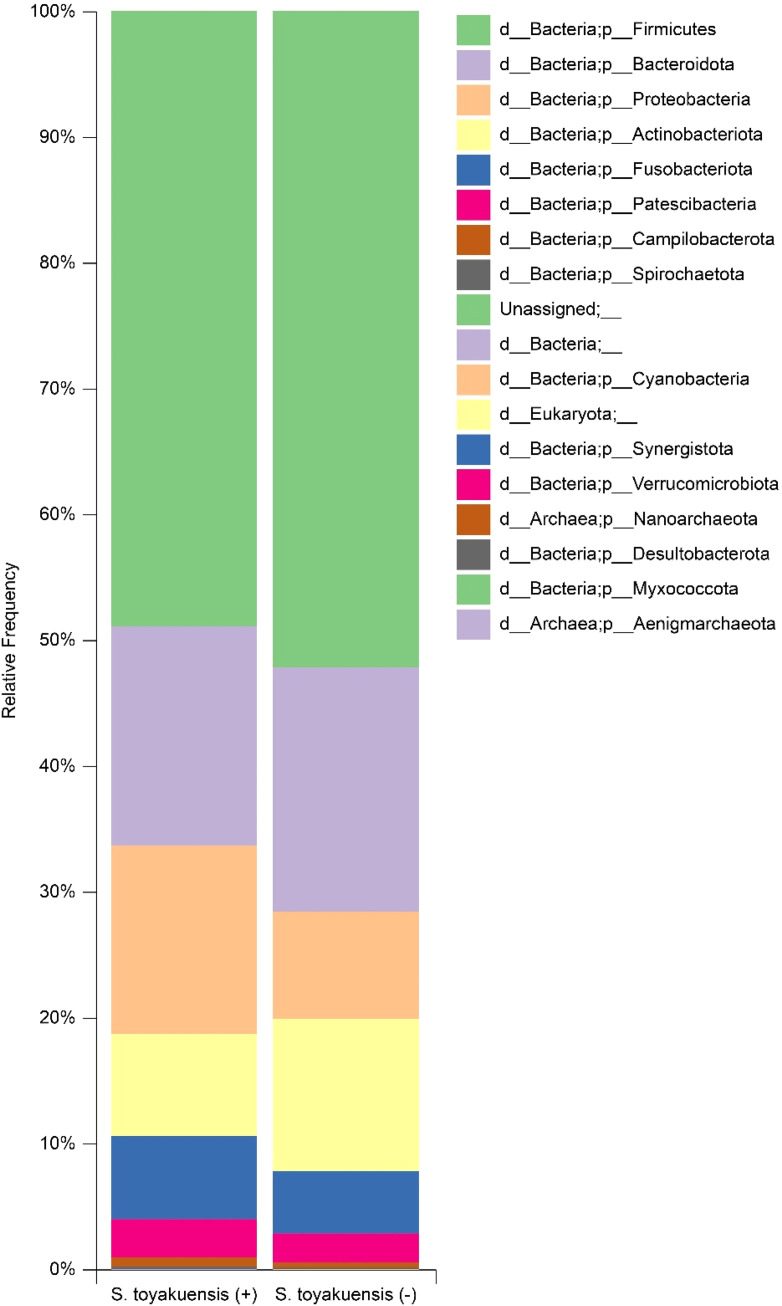
Taxonomic composition of salivary microbiomes in S. toyakuensis (+) and (-) groups. Bar plots showing the relative abundances of microbial phyla in saliva samples from S. toyakuensis (+) and (-) groups. Firmicutes and Bacteroidetes were the dominant phyla in both groups, with variations in the proportions of other phyla such as Proteobacteria, Actinobacteriota, and Fusobacteriota.

As previously reported, a total of 10,803,843 reads were obtained after quality filtering across 60 saliva samples, with an average of 180,064.05 reads per sample (range: 120,719-203,022).^9^ Similarly, 3,724 amplicon sequence variants were identified. Taxonomic analysis confirmed the presence of 18 microbial phyla, 25 classes, 63 orders, 104 families, 234 genera, and 493 species. In *S. toyakuensis* (+) samples, the most abundant microbial phyla were *Firmicutes* (48.9%) and *Bacteroidetes* (17.4%), followed by *Proteobacteria* (15.1%), *Actinobacteriota* (8.1%), *Fusobacteriota* (6.6%), *Patescibacteria* (3.0%), and *Campylobacterota* (0.7%). In *S. toyakuensis* (−) samples, the dominant phyla were *Firmicutes* (52.2%) and *Bacteroidetes* (19.4%), followed by *Actinobacteriota* (12.1%), *Proteobacteria* (8.5%), *Fusobacteriota* (4.9%), *Patescibacteria* (2.3%), and *Campylobacterota* (0.4%) as shown in Figure 2.

**Fig. 3:**
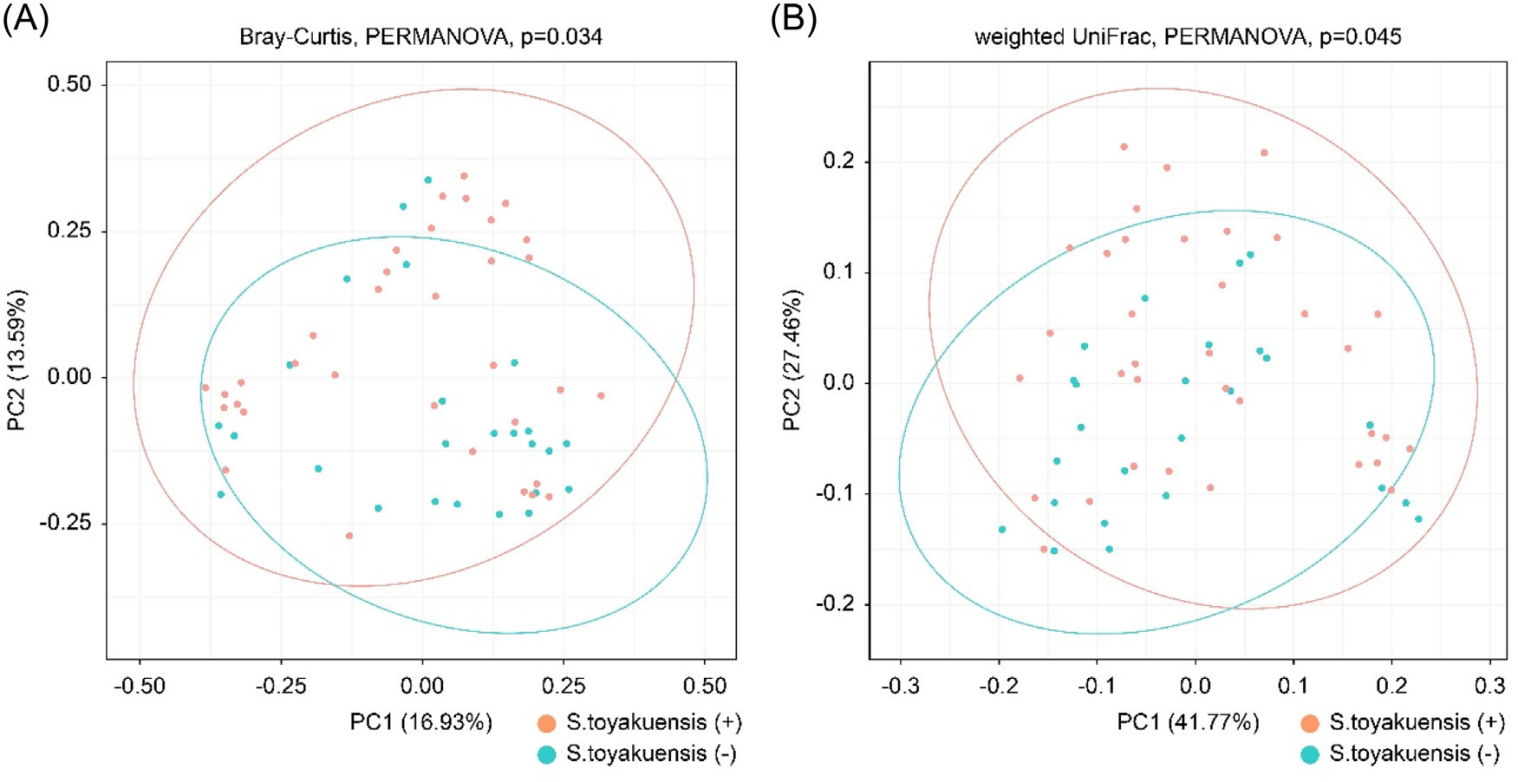
Microbial community composition in S. toyakuensis (+) and (-) groups. Principal Coordinate Analysis (PCoA) plots showing distinct clustering of salivary microbiomes between S. toyakuensis (+) and (-) groups. Pairwise PERMANOVA tests confirm significant differences in microbial composition based on Bray-Curtis and weighted UniFrac distances (*P = .*034 and *P = .*045, respectively).

The Kruskal-Wallis test revealed no significant difference in alpha diversity—measured by the Shannon, Faith’s phylogenetic diversity, observed features, and evenness indices—between the *S. toyakuensis* (+) and *S. toyakuensis* (−) groups (*P > .*05). The PCoA plots (Figure 3 A and B) illustrate the distinct clustering of salivary microbiomes according to *S. toyakuensis* status. This clustering was further supported by pairwise PERMANOVA tests of Bray-Curtis (*P = .*034) and weighted UniFrac (*P = .*045) distances.

**Fig. 4:**
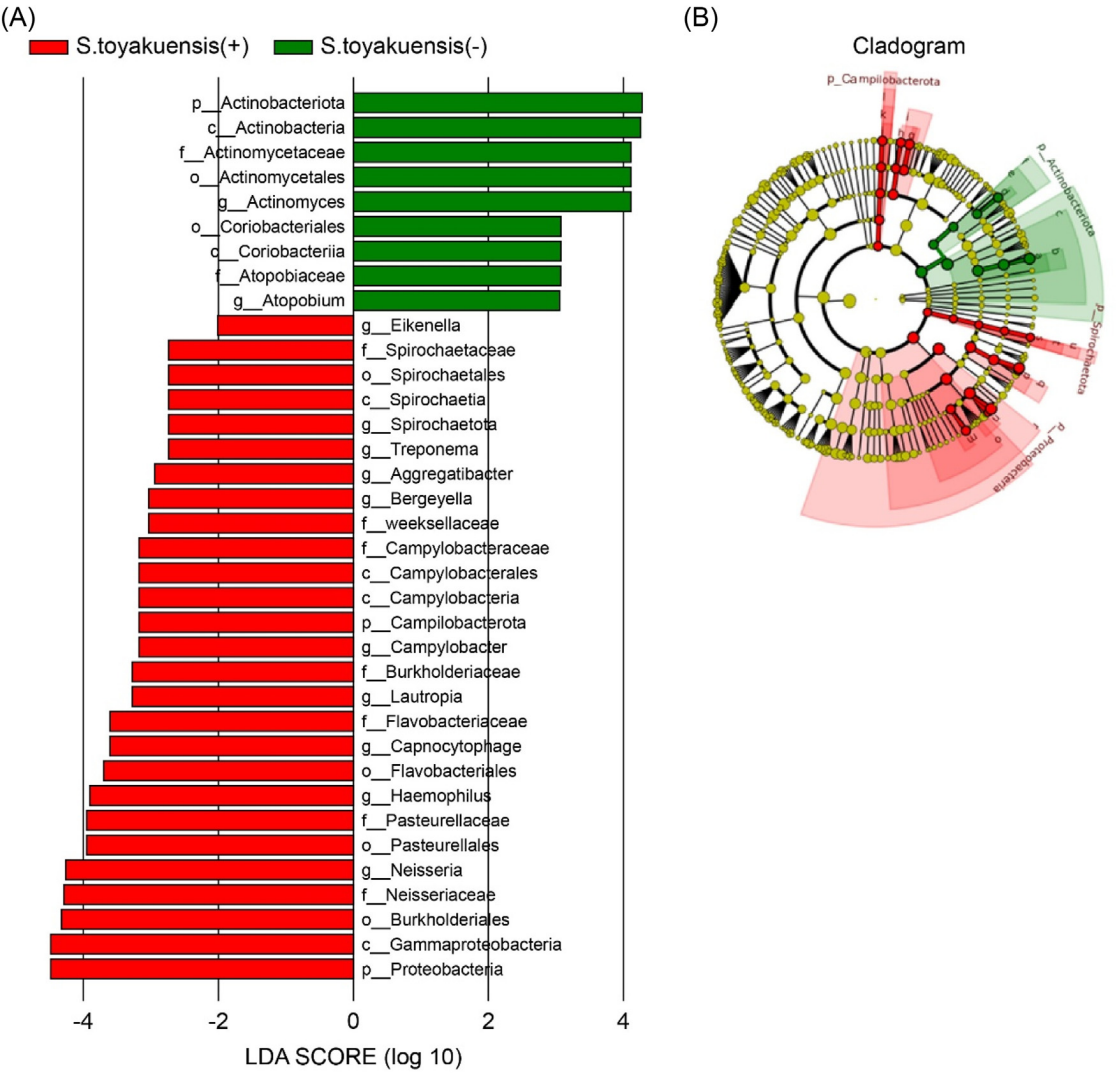
Differentially abundant taxa in S. toyakuensis (+) and (-) groups. (A) LDA score bar plot highlighting taxa with significant differences between groups (LDA score ≥ 2), showing enrichment of specific genera in each group. (B) Phylogenetic cladogram illustrating evolutionary relationships among differentially abundant taxa, highlighting distinct microbial compositions between S. toyakuensis (+) and (-) groups.

LEfSe analysis revealed that the salivary microbial composition of the *S. toyakuensis* (+) group demonstrated a significant dominance of several genera, each comprising more than 1% of the total microbial community: *Neisseria* (9.56% vs. 5.89%, *P = .*045, LDA = 4.99), *Haemophilus* (3.81% vs. 2.00%, *P = .*029, LDA = 4.58), *Campylobacter* (0.72% vs. 0.44%, *P = .*025, LDA = 3.87), and *Capnocytophaga* (1.49% vs. 0.70%, *P = .*003, LDA = 4.16). Conversely, the *S. toyakuensis* (−) group was characterized by a significant dominance of *Actinomyces* (5.76% vs. 2.98%, *P = .*027, LDA = 4.76). Taxa with LDA scores (log10) of 2 or higher are illustrated in the LDA score bar plot (Figure 4A), which highlights distinct microbial differences between the groups. Subsequently, Figure 4B provides an evolutionary perspective, demonstrating the predominance of *Campylobacterota, Spirochaete*, and *Proteobacteria* in the detection group, compared with the predominance of *Actinobacteriota* in the non-detection group.

**Fig. 5:**
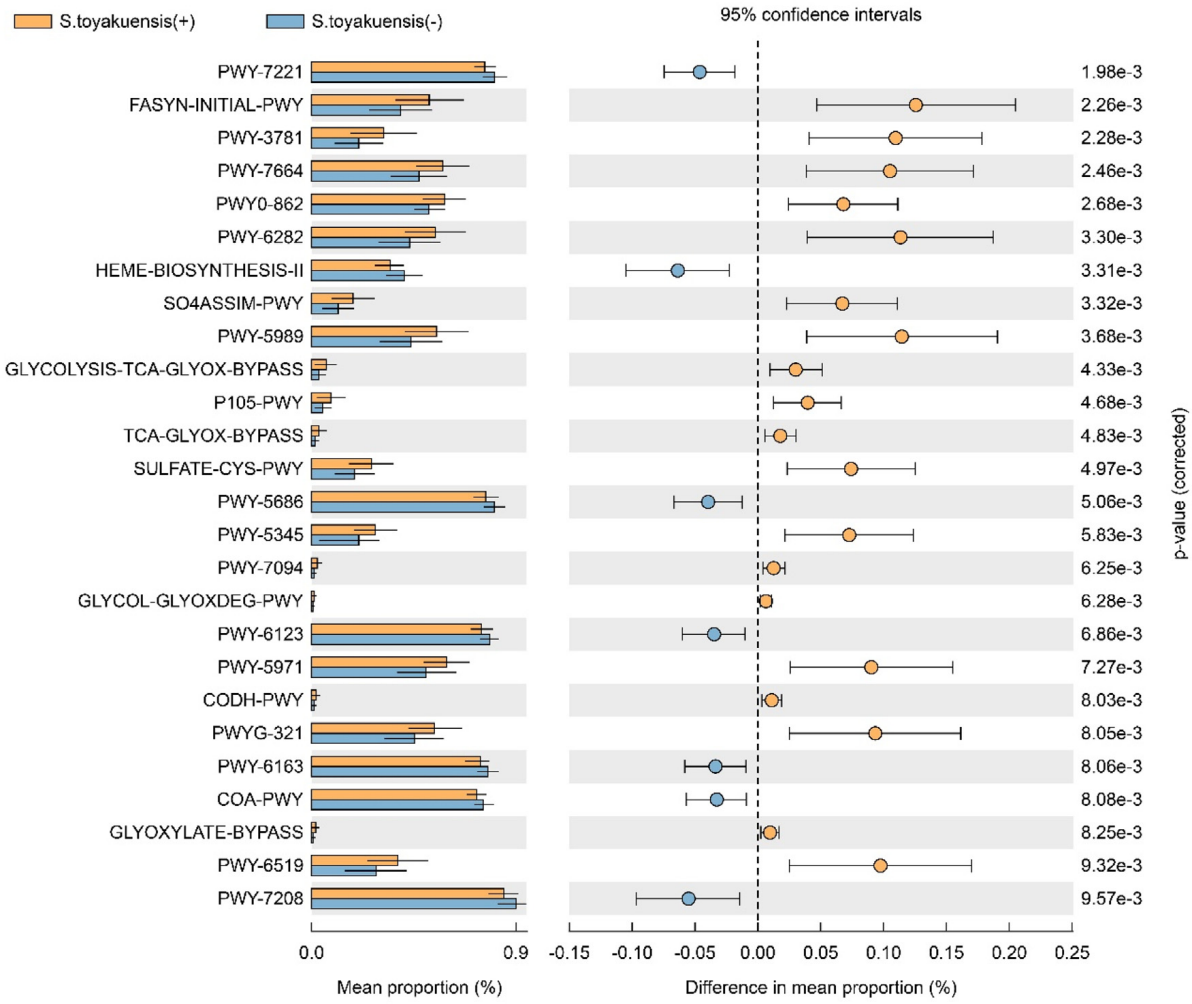
Predicted functional pathway differences in salivary microbiota between S. toyakuensis (+) and (-) groups. Functional profiles predicted by PICRUSt and analyzed using STAMP reveal statistically significant differences in 26 pathways between the groups.

The functional profiles predicted by PICRUSt2 and analyzed using STAMP are shown in Figure 5, which highlights significant differences in 26 metabolic pathways between the detected and non-detected groups. In the *S. toyakuensis* (+) group, 19 pathways—including the glyoxylate cycle, mycolate biosynthesis, assimilatory sulfate reduction I, the superpathway of L-methionine biosynthesis, and the superpathway of glycol metabolism and degradation—exhibited significantly higher mean proportions (*P < .*01), suggesting activation of these pathways. Conversely, seven pathways—including heme biosynthesis II, chorismate biosynthesis from 3-dehydroquinate, the purine biosynthesis pathway, and the pyrimidine nucleobase salvage superpathway—showed significantly lower mean proportions (*P < .*01), indicating that these pathways were likely downregulated in the detected group.

## Discussion

This study is the first to demonstrate the presence of *S. toyakuensis*, a recently identified multidrug-resistant bacterium,^1^^,^^7^ in human saliva. Using 16S rRNA NGS, saliva samples from 60 healthy young individuals were analyzed. *S. toyakuensis* was detected in 35 participants (58.3%). Although the relative abundance was low, with a median value of 0.0016%, a consistent presence within the salivary microbiome was observed. No significant differences were found between the *S. toyakuensis* (+) and *S. toyakuensis* (−) groups in terms of demographic characteristics or oral environmental factors, including age, sex, stimulated salivary secretion rate, salivary pH, buffering capacity, number of teeth, The Plaque Control Record score, oral bacterial count, or the presence of oral *Candida species*. Host age is a recognized factor influencing the structure of the oral microbiome, potentially mediated by physiological changes such as increased salivary flow during puberty.^11^^,^^12^ Although this study did not perform detailed age adjustment, no significant difference in salivary flow was observed between the *S. toyakuensis* (+) and *S. toyakuensis* (−) groups. Future studies should incorporate comprehensive analyses adjusted for age and sex to better understand the microbial dynamics.

The Oral microbiota composition is highly susceptible to dietary factors, such as the overconsumption of fermentable carbohydrates like sucrose, which is widely considered a major determinant causing in-vivo shifts in oral biofilm.^4^^,^^13^ Since this study did not collect detailed dietary data from participants, the influence of nutritional habits on the observed distribution and functional profiles of *S. toyakuensis* cannot be assessed.

*Candida* species are widely recognized as indicators of oral hygiene and are key participants in cross-kingdom interactions, notably with cariogenic bacteria like Streptococcus mutans.^14^^,^^15^ This interaction is mediated by metabolites (e.g., farnesol) which stimulate *S. mutans* growth and alter biofilm architecture, synergizing the virulence of plaque biofilms. Since the 16S rRNA gene sequencing (V3-V4 region) used for bacterial profiling cannot detect fungi, we employed a separate culture-based method using specialized media (Colorex Candida plates) to assess *Candida* prevalence. Nonetheless, our analysis found no significant association between *Candida* detection and the presence of *S. toyakuensis*. Studies summarized by Wade et al. have indicated that oral microbiome composition can be influenced by factors such as immune dysfunction, reduced salivary flow, excessive sugar intake, periodontitis, and diabetes.^16^ Further studies are planned to explore other factors potentially associated with *S. toyakuensis* colony formation.

Sequencing analysis of the 16S rRNA V3-V4 region identified 493 microbial species in all samples. Esberg et al.^17^ reported that Oxford Nanopore Technology and Illumina MiSeq analyses of the same region detected 499 and 457 microbial species, respectively, in healthy saliva sample. These results are consistent with those of previous studies. In both groups, *Firmicutes* and *Bacteroidetes* were the dominant phyla. However, *Proteobacteri*a were more abundant than *Actinobacteriota* in the detection group, whereas the reverse trend was observed in the non-detection group. No significant differences in alpha diversity analysis were detected between the groups, suggesting that the presence of *S. toyakuensis* did not substantially affect individual salivary microbiome diversity. Beta diversity analysis showed that the diversity predominantly increased in the *S. toyakuensis* (+) group compared with that in the *S. toyakuensis* (−) group; therefore, bacterial species exhibiting increases or decreases were specifically examined.

LEfSe analysis identified notable differences in the distribution of microbial genera between the groups. In the *S. toyakuensis* (+) group, genera such as *Neisseria, Haemophilus, Campylobacter*, and *Capnocytophaga*, which are commonly found in the respiratory and oral microbiota, accounted for more than 1% of the microbiome. Conversely, the *S. toyakuensis* (−) group exhibited a significant predominance of *Actinomyces* (5.76% vs. 2.98%, *P = .*027). At the phylum level, *Campylobacterota, Spirochaetota*, and *Proteobacteria* were predominant in the detection group, whereas *Actinobacteriota* were more prevalent in the non-detection group. These findings imply that the detection group harbored higher populations of anaerobic or microaerophilic bacteria, whereas the non-detection group was dominated by sugar-metabolizing bacteria. Thus, the colonization of *S. toyakuensis* may alter the oral environment and promote the prevalence of microbial groups with distinct evolutionary backgrounds. Functional pathway analysis revealed that *S. toyakuensis* influenced the functional diversity of the salivary microbiome. Notably, 19 metabolic pathways, including the glyoxylate cycle, mycolate biosynthesis, assimilatory sulfate reduction I, L-methionine biosynthesis superpathway, and the carbohydrate metabolism/degradation superpathway, were activated in the detection group. These pathways are involved in energy metabolism and nutrient acquisition, likely supporting bacterial survival and adaptation in the oral environment. Conversely, seven metabolic pathways—such as heme biosynthesis II, chorismate biosynthesis from 3-dehydroquinate, purine biosynthesis, and the pyrimidine nucleobase salvage superpathway—were deactivated. These pathways are essential for cellular growth and DNA synthesis, suggesting that the genetic activity and metabolic processes of the salivary microbiome are altered. The study by Ma et al.^18^ demonstrated that periodontitis significantly alters both the microbial composition and functional gene profiles of the oral microbiome, supporting the validity of PICRUSt-based functional prediction. In our study, the presence of *S. toyakuensis* and shifts in specific functional pathways may reflect subtle ecological changes and host health status. Given that certain oral bacteria can contribute to systemic diseases, and systemic conditions may in turn reshape the oral microbiome, these findings suggest a potential bidirectional relationship. The possible link between *S. toyakuensis* and antimicrobial resistance within this context warrants further investigation.

## Limitation

This study used the NGS targeting 16S rRNA gene to detect *S. toyakuensis* and investigate its distribution and potential influence on the salivary microbiome. Although the findings provide new insights into the presence and microbial dynamics of this novel organism, several limitations must be considered.

First, Although *S. toyakuensis* was initially proposed as a novel species, a comprehensive genomic analysis reported by Kilian et al.^19^ suggests its reclassification as a later heterotypic synonym of *S. mitis*. This situation arises because the strain shares such high similarity (the type strain shares 99.5% 16S rRNA gene sequence identity with S. mitis NCTC 12261) with closely related strains belonging to the *S. mitis* Lineage, forming a continuum of pairwise similarities. Consequently, accurate species-level identification is difficult to achieve solely based on 16S rRNA gene sequence analysis, as this method is limited in its ability to resolve phylogenetically close species within this group.

In this study’s analysis, a specific mutation was identified near the 380 bp region of the 16S rRNA gene through multiple alignment of sequences from the RefSeq database, and this variation served as the basis for assigning the representative sequence as the top hit for *S. toyakuensis* (Sup.Figure 1). However, considering the biological reality of the genomic diversity continuum within the *S. mitis* group, this single mutation is not a definitive basis for determining species boundaries. As a result, the findings indicate the potential presence of *S. toyakuensis* but cannot definitively confirm its identification. Ideally, specific primers designed for PCR or quantitative PCR (qPCR) would enable more definitive identification of *S. toyakuensis*. However, technical challenges in primer design made these approaches infeasible.

Furthermore, functional pathway analysis was based on predictive metagenomics (PICRUSt2), which relied on reference databases. Therefore, the predicted functional profiles may not accurately reflect actual metabolic activity. Future studies should incorporate metatranscriptomic and metabolomic analyses to provide direct evidence for functional changes.

In previous studies, *S. toyakuensis* was identified based on a single isolate, and its antimicrobial resistance characteristics were analyzed.^7^ However, whether other bacteria belonging to the same species exhibit similar resistance profiles remains unclear. Further evidence is required to determine whether *S. toyakuensis* possesses multidrug resistance. Genetic analyses and phenotypic testing of multiple isolates are necessary to assess the prevalence of resistance and its impact on genetic variation. This study relied on DNA-based data extracted from saliva samples and did not include culture-based susceptibility testing. This limitation highlights the need for further research to confirm the antimicrobial resistance potential of *S. toyakuensis*.

Additionally, another notable limitation of this study is the sample size. Due to financial constraints, the analysis was conducted with 35 participants in the *S. toyakuensis*-positive group and 25 in the negative group. This number falls short of the recommended sample size for certain statistical analyses, such as alpha diversity and functional pathway comparisons, which typically require approximately 64 participants per group to detect moderate effect sizes with sufficient statistical power (80% at α = 0.05). As a result, the statistical power of the study may be limited, and some potentially meaningful differences may not have reached significance. Therefore, the findings should be interpreted as exploratory and hypothesis-generating rather than conclusive. Future studies with larger cohorts are needed to validate these results and further investigate the ecological and clinical significance of *S. toyakuensis* in the oral microbiome.

The participants in this study were recruited from a dental department, which may have introduced selection bias. Patients seeking dental care may have different oral health profiles compared with the general population, potentially limiting the generalizability of the findings. The oral microbiome characteristics observed in this study may not accurately represent individuals with different health conditions or those who do not routinely visit dental clinics.

Considering these limitations, this study serves as an initial investigation into *S. toyakuensis* and its potential role in the salivary microbiome. However, further analyses involving strain isolation, broader population sampling, and comprehensive functional analyses are essential to clarify its contribution to antimicrobial resistance and its interactions within the oral microbial community.

## Conclusion

This study demonstrates that *S. toyakuensis* can colonize the salivary microbiome of healthy young individuals and may influence the composition and functional characteristics of the oral microbiome. The relatively high prevalence of this potentially multidrug-resistant bacterium in the oral microbiome of healthy individuals highlights its significance as a potential public health concern. Further research is needed to elucidate its ecological role and impact on the oral environment.

## Author contributions

**Nami Obayashi**: Formal analysis, investigation, writing – original draft, visualization, and funding acquisition. **Tomoaki Shintani**: Funding acquisition, methodology, investigation, resources, writing, review, and editing. **Nagisa Morihara**: Software, formal analysis, and visualization. **Miki Kawada-Matsuo**: Supervision and conceptualization. **Toshinori Ando**: Investigation and writing – review and editing. **Rie Miyata**: Data curation. **Yuka Hayashi**: Investigation and resources. **Nanako Kataoka**: Investigation. **Kotaro Tanimoto**: Data collection. **Hiroyuki Kawaguchi**: Writing – review and editing. **Hitoshi Komatsuzawa**: Supervision, conceptualization, methodology, and writing – review and editing. **Mikihito Kajiya**: Project administration and writing – review and editing. All authors have read and agreed to the final version of the manuscript.

## Conflict of interest

The authors declare that they have no competing interests.

## Glossary

Alpha diversity: A measure of microbial richness and evenness within a sample, assessed using indices such as Shannon or Faith’s phylogenetic diversity.
Beta diversity: A comparison of microbial community composition between groups or samples, using distance metrics like Bray-Curtis and UniFrac.
Bray-Curtis / UniFrac: Statistical distance measures used to assess dissimilarity between microbial communities.
Candida species: A genus of yeast organisms, some of which are part of the normal oral flora but may overgrow under certain conditions
Dielectrophoretic impedance: A technique for estimating bacterial concentrations by detecting electrical impedance changes.
Faith’s phylogenetic diversity: An alpha diversity metric incorporating phylogenetic relationships among species in a sample.
Functional pathway: A series of enzymatic steps that contribute to a specific metabolic function within a microbial community.
Glyoxylate cycle: A metabolic pathway enabling organisms to convert fats into sugars, bypassing decarboxylation steps of the TCA cycle.
Interquartile range (IQR): A statistical measure representing the spread between the 25th and 75th percentiles.
LDA score: A numerical value indicating how strongly a feature (e.g., bacterial taxon) differentiates between groups in LEfSe analysis.
MetaCyc: A curated database of experimentally determined metabolic pathways across different organisms.
Metabolomics: The study of small-molecule metabolites in biological samples, reflecting active biochemical processes.
Metatranscriptomics: The sequencing and analysis of RNA transcripts in a microbial community to assess real-time gene expression.
Mycolate biosynthesis: A lipid-synthesis pathway relevant to the cell envelope structure of certain bacteria, such as mycobacteria.
Naïve Bayesian classifier: A statistical algorithm used in QIIME2 for taxonomic assignment of DNA sequences.
Observed features: A count of the unique microbial taxa observed in a sample; a simple measure of richness.
PERMANOVA: A non-parametric test to evaluate differences in multivariate data, such as microbial community composition.
PICRUSt2: A bioinformatics tool that predicts the functional capabilities of microbial communities based on 16S rRNA gene sequences. It uses phylogenetic placement to infer gene content from reference genomes, enabling estimation of metabolic pathways such as those cataloged in KEGG or MetaCyc, without requiring whole metagenome sequencing.
QIIME2: Quantitative Insights into Microbial Ecology 2; an open-source platform for microbiome data analysis.

## Appendix Supplementary materials

Image, application 1

## Footnotes

Supplementary material associated with this article can be found in the online version at doi:10.1016/j.identj.2025.103987.

## References

[1] Naghavi M., Vollset S.E., Ikuta K.S. (2024). Global burden of bacterial antimicrobial resistance 1990-2021: a systematic analysis with forecasts to 2050. Lancet.

[2] Tackling drug-resistant infections globally: final report and recommendations the review on antimicrobial resistance chaired by Jim O’Neill. 2016.

[3] Carr V.R., Witherden E.A., Lee S. (2020). Abundance and diversity of resistomes differ between healthy human oral cavities and gut. Nat Commun.

[4] Anderson A.C., von Ohle C., Frese C. (2023). The oral microbiota is a reservoir for antimicrobial resistance: resistome and phenotypic resistance characteristics of oral biofilm in health, caries, and periodontitis. Ann Clin Microbiol Antimicrob.

[5] Peng X., Cheng L., You Y. (2022). Oral microbiota in human systematic diseases. Int J Oral Sci.

[6] Nishihama S., Kawada-Matsuo M., Le M.N.T. (2025). Oral colonization of antimicrobial-resistant bacteria in home health care participants and their association with oral and systemic status. Sci Rep.

[7] Wajima T., Hagimoto A., Tanaka E., Kawamura Y., Nakaminami H. (2022). Identification and characterisation of a novel multidrug-resistant streptococcus, Streptococcus toyakuensis sp. nov., from a blood sample. J Glob Antimicrob Resist.

[8] Munson E., Carella A., Carroll K.C. (2023). Valid and accepted novel bacterial taxa derived from human clinical specimens and taxonomic revisions published in 2022. J Clin Microbiol.

[9] Shintani T., Obayashi N., Yoshimoto T. (2025). Effect of stimulated salivary volume on dysbiosis of the salivary microbiome in children and young adults. Int Dent J.

[10] O’Leary T.J., Drake R.B., Naylor J.E. (1972). The plaque control record. J Periodontol.

[11] Takeshita T., Kageyama S., Furuta M. (2016). Bacterial diversity in saliva and oral health-related conditions: the hisayama study. Sci Rep.

[12] Crielaard W., Zaura E., Schuller A.A., Huse S.M., Montijn R.C., Keijser B.J.F. (2011). Exploring the oral microbiota of children at various developmental stages of their dentition in the relation to their oral health. BMC Med Genomics.

[13] Anderson A.C., Rothballer M., Altenburger M.J. (2018). In-vivo shift of the microbiota in oral biofilm in response to frequent sucrose consumption. Sci Rep.

[14] Metwalli K.H., Khan S.A., Krom B.P., Jabra-Rizk M.A. (2013). Streptococcus mutans, Candida albicans, and the human mouth: a sticky situation. PLoS Pathog.

[15] Kim D., Sengupta A., Niepa T.H.R. (2017). Candida albicans stimulates Streptococcus mutans microcolony development via cross-kingdom biofilm-derived metabolites. Sci Rep.

[16] Wade WG. (2021). Resilience of the oral microbiome. Periodontol 2000.

[17] Esberg A., Fries N., Haworth S., Johansson I. (2024). Saliva microbiome profiling by full-gene 16S rRNA Oxford Nanopore Technology versus Illumina MiSeq sequencing. NPJ Biofilms Microbiomes.

[18] Ma Z., Jiang Z., Dong H. (2024). Microbial communities and functional genes in periodontitis and healthy controls. Int Dent J.

[19] Kilian M., Slotved H.C., Fuursted K., D’mello A., Tettelin H. (2025). Re-evaluation of boundaries of Streptococcus mitis and Streptococcus oralis and demonstration of multiple later synonyms of Streptococcus mitis, Streptococcus oralis and Streptococcus thalassemiae: description of Streptococcus mitis subsp. carlssonii subsp. nov. and emended description of Streptococcus mitis. Int J Syst Evol Microbiol.

