# Supplementary material for: Prevalence and Ecological Role of Streptococcus toyakuensis in Saliva of Healthy Young Individuals
Source: Int Dent J. 2025 Nov 9;76(1):103987. doi: 10.1016/j.identj.2025.103987 (PMC12648603; doi:10.1016/j.identj.2025.103987)
Supplement: Supplementary file 1 [file mmc1.docx]

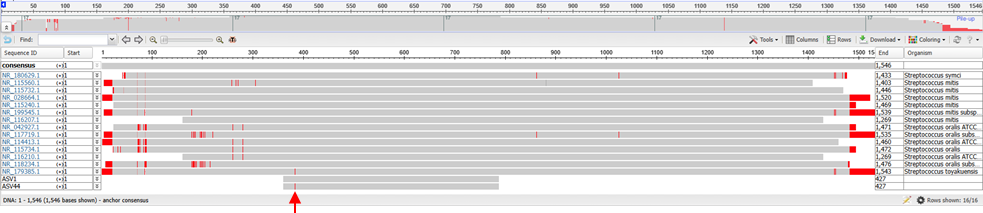


**Supplementary Figure 1. Multiple sequence alignment of S. toyakuensis and related species based on 16S rRNA gene sequences**
Reference sequences of *S. toyakuensis*, *S. mitis*, *S. oralis*, and other closely related species from the RefSeq database were aligned with representative sequences assigned to *S. toyakuensis* and *S. mitis* using QIIME2. The alignment was visualized using a sequence viewer. ASV44 corresponds to *S. toyakuensis*, and a species-specific mutation was observed near base position 380 (indicated by a red arrow). This mutation may serve as a distinguishing feature for identifying *S. toyakuensis* among closely related taxa.
